# Temporomandibular Disorders: Management of Diagnostics and Therapy in the Context of Orthodontic Treatment—A Survey Among German Orthodontists

**DOI:** 10.3390/dj13040167

**Published:** 2025-04-17

**Authors:** Tobias Klur, Sara Portegys, Isabelle Graf, Sven Scharf, Bert Braumann, Teresa Kruse

**Affiliations:** Department of Orthodontics, Faculty of Medicine and University Hospital Cologne, University of Cologne, 50931 Cologne, Germany; tobias.klur@uk-koeln.de (T.K.); sara.portegys@uk-koeln.de (S.P.); isabelle.graf@uk-koeln.de (I.G.); s.scharf@kfo-scharf.de (S.S.); bert.braumann@uk-koeln.de (B.B.)

**Keywords:** temporomandibular disorder, orthodontic therapy, TMD, orthodontic treatment, temporomandibular joint clicking, functional occlusal factors, TMD screening

## Abstract

**Background/Objectives**: To evaluate the role of temporomandibular disorder (TMD)-related diagnostics in orthodontic treatment routines and investigate what consequences are drawn from symptoms concerning orthodontic treatment planning and therapy. **Methods**: All officially listed orthodontists in Germany were surveyed about their professional background, TMD-related specialization, and concrete clinical procedures. Anonymized responses were systematized, manually checked, and statistically analyzed. Differences in reported TMD-related procedures depending on orthodontists’ professional experience and specialization were determined using Fisher’s exact tests. **Results**: A total of 2359 questionnaires were sent out, of which 630 could be evaluated. The majority of the orthodontists surveyed stated that they perform either a brief TMD screening or a complete functional analysis. In total, 21.1% of the respondents base their examination on the patient’s medical history. A second complete functional analysis is performed by 33% of the responding orthodontists during the course of orthodontic therapy, and by 56.6% only in the case of an initial pathological finding. For 60.1% of the respondents, pre-therapeutically diagnosed, non-painful temporomandibular joint clicking has an influence on orthodontic treatment planning. Only 4.3% of respondents take no further action prior to orthodontic therapy in the case of TMD symptoms. There is an indication that professional experience has no influence on the procedure, whereas a specialization in the field of TMDs does. **Conclusions**: A discrepancy between the current state of research and standard procedures in German orthodontic practices may lead to an overly detailed examination. However, this has no health disadvantages for the patient.

## 1. Introduction

Temporomandibular disorders (TMDs) are a relevant public health problem affecting up to 34% of the worldwide population [1]. As a subtype of craniofacial pain, it describes a painful or dysfunctional clinical diagnosis that affects the masticatory musculature, the temporomandibular joint (TMJ) and associated structures, or both. The expanded taxonomy of TMDs, which is used internationally, consists of four categories: temporomandibular joint disorders, masticatory muscle disorders, headache disorders, and disorders affecting associated structures [2]. A limitation or deviation in the mandibular range of motion, TMJ clicking, and/or headaches and facial pain are further symptoms [3,4,5]. The etiology of the described pathology is multifactorial. Thus, in recent decades, various factors have been discovered, discussed, and weighted differently, including macro-trauma, micro-trauma, and psychosocial, genetic, hormonal, and other systemic factors [4,5]. Historically, TMDs have been associated with dental malocclusions. In the 1970s and 1980s, static and dynamic occlusion were assigned a predominant role in the occurrence of symptoms [6,7,8]. Various clinical studies have demonstrated that this influencing factor is of minor impact [9].

Compared with the general population, the prevalence of TMDs in patients seeking orthodontic treatment is much higher and ranges from 21.1% to 73.3% [5,10]. Orthodontics addresses the detection and/or prevention of teeth and jaw anomalies, ensures an undisturbed development of the stomatognathic system, and aims to correct dentofacial anomalies. A traditional objective is to improve static and dynamic occlusion—and thus masticatory function. It has been shown that a class II occlusion, a unilateral crossbite, and an unstable occlusal relationship or the presence of a lateral forced bite are strong risk factors for the development of TMDs [5,11,12]. The (temporary) elimination of interfering occlusal factors by means of an occlusal splint may reduce the existing pain [13,14]. Along these lines, orthodontic treatment also influences the development or progression of TMDs. However, to date, no valid data are available that clearly show a connection between the changed occlusion after orthodontic treatment (dynamic or static) and the development of TMDs. Various reviews or epidemiological studies found no or only weak correlations [15,16,17]. No direct causal relationship between orthodontic therapy and TMDs could be shown, and triggering or mitigating factors could not be identified [15,18,19].

A routine TMD-related examination prior to orthodontic therapy seems to be crucial, and not only for forensic reasons. A brief screening is generally recommended as part of orthodontic treatment planning in order to exclude the presence of TMD signs or symptoms and to treat it prior to an upcoming orthodontic treatment. In addition, asymptomatic findings that have not (yet) been clinically relevant to date, such as compression forces on the TMJ, may be detected at an early stage. Whether such compensated (anatomical) abnormalities may influence subsequent orthodontic treatment and consequently need to be considered in orthodontic treatment planning is still unclear [20].

In national and international clinical practice, many different examination protocols have been established for the evaluation of a possibly present TMD. Such an examination protocol should evaluate at least the following aspects: mandibular movements, the stomatognathic musculature, including the cervical musculature, the temporomandibular joints and their limiting structures, and static and dynamic occlusion. In addition, inquiring about psychogenic components is recommended [21,22]. When choosing a protocol, high sensitivity and high specificity play a very important role, as well as high inter-rater reliability. Both the Diagnostic Criteria for Temporomandibular Disorders (DC/TMD) protocol and the Research Diagnostic Criteria for Temporomandibular Disorders (RDC/TMD) protocol show high values regarding these three required properties [2,23].

The investigation of a possible TMD often takes a lot of time for the practitioner; therefore, some clinicians propagate that symptomless patients who are about to undergo extensive dental rehabilitation or orthodontic treatment should first be examined with a brief examination or screening with regard to functional abnormalities. A complete functional analysis is then recommended only in the case of positive findings. The screenings naturally take much less time and are therefore much less cost-intensive. For example, in the screening form of the German Society for Functional Diagnostics and Therapy (DGFDT), the patient is first asked about pain and problems with opening their mouth. Five parameters are then examined and documented with simple yes/no answers. Depending on the results, extended diagnostics are recommended. The German Pain Society recommends the following examinations as minimal diagnostics when a TMD is suspected: pain-related history, whole-body drawing, recording of psychogenic components such as stress or depression, and a panoramic radiograph [24]. For further diagnosis and/or to provide appropriate management, adjunctive dental or medical imaging (such as magnetic resonance imaging) may be required [22].

The aim of this study was to investigate the relevance of TMJ examinations in routine orthodontic treatment, taking into account the professional experience and possible specialization of orthodontists. The main aim was to analyze the general handling and timing of functional diagnostic measures and their consequences for routine orthodontic treatment. The focus of this study was to investigate the potential heterogeneity in the management of diagnostics and therapy without going into the details of the diagnostics or therapy itself and without focusing on patients who seek orthodontic treatment due to TMDs. With the help of a nationwide survey of all officially listed orthodontists in Germany, a representative overview of the frequency, timing, and consequences of TMJ examinations in private practice in a European country was obtained.

## 2. Materials and Methods

This study was approved by the Ethics Committee of the Medical Faculty of the University of Cologne (approval number: 20-1099, approval date: 7 July 2020). All orthodontic specialist practices listed on the official websites of dental associations in Germany were included in the survey. Data were collected using a questionnaire sent by mail. The questionnaires were sent out in August 2020. All responding participants provided written informed consent. Responses given within a specified inclusion period of 3 months were taken into account.

The results of the returned questionnaires were entered into Microsoft Excel 2016 using the readout program Remark Office version 14.0 and reviewed manually. The questionnaire contained 8 items addressing respondents’ professional background, specialization in the field of TMDs, and specific clinical procedures (Appendix A). Ambiguous or non-evaluable answers were treated as non-responses.

The total sample consisted of 2359 possible participants. In total, 27 of the 2359 questionnaires sent out were returned as undeliverable. A total of 630 questionnaires were completed, returned, accepted, and evaluated within the three-month inclusion period, resulting in a response rate of 27%. Due to a few individual responses that could not be evaluated, the resulting size of the analytical sample varied between n = 605 and n = 627, depending on the question. A sample size calculation indicates that, under the assumption of small to moderate differences between groups (i.e., a standardized effect size of 0.2), a sample of n = 325 would be sufficient to detect such differences, which our sample substantially exceeds.

Statistical analyses were performed using IBM SPSS version 16.0.0.1. Missing data were handled using case-wise deletion. Missing data were assumed to be missing completely at random (MCAR). Little’s test provided no evidence to reject the MCAR hypothesis (test statistic = 53.4, *p*-value = 0.497). Descriptive analyses of metric characteristics were carried out using means and standard deviations. Categorical variables were descriptively assessed using absolute and relative frequencies. For inferential statistical analysis, some variables were dichotomized, identifying orthodontists with less/more than 25 years of professional experience and orthodontists with/without specialization in the field of TMDs. The categorization into less than 25 years since licensure (equated with professional experience) and 25 or more years results from the calculated median split. Differences in the responses evaluated between these groups were examined using Fisher’s exact tests. To ensure robustness, analyses were repeated using the chi-squared test and Yates’ corrected chi-squared test. As the results remained consistent across methods, the results of Fisher’s exact tests are reported. Group size differences were reported using odd ratios (including their confidence intervals). A difference was considered statistically significant at a *p*-value of less than 0.05.

## 3. Results

The responding orthodontists had been in private practice for a mean of 17.64 ± 9.69 years. The professional experience measured since the year of licensure was 25.26 ± 9.44 years. For the majority of respondents, their practice had neither a focus on TMD diagnostics/therapy (n = 474, 76.3%), nor did the orthodontist possess a corresponding additional qualification (n = 462, 73.8% without additional qualifications).

### 3.1. Diagnostics Before Orthodontic Therapy

A total of 67% of the respondents (n = 422) stated that they always obtain a brief physical examination corresponding to screening/minimal diagnostics prior to orthodontic therapy. A total of 15.7% (n = 99) stated that they always perform a complete functional analysis prior to therapy, while 36.8% (n = 232) of the participants who responded to the questionnaire stated that they only did so if an initially performed screening was conspicuous. In total, 21.1% of respondents (n = 133) reported performing TMD screening only if the patient expressed TMD symptoms during the interview or in the medical history. Furthermore, 13.8% (n = 87) stated that they always refer patients to a colleague who is specialized in the TMJ for further diagnostics if the patient’s history and/or screening were positive. A total of 2.5% of respondents (n = 16) indicated that they did not perform screening or any other examination of the TMJ prior to orthodontic treatment.

Frequently, response options were combined. For example, 2.9% of the respondents (n = 18) stated that they conducted both screening and a thorough physical examination before orthodontic therapy. A total of 22.5% (n = 144) indicated that they initially performed a screening and only a complete functional analysis if the initially performed screening was conspicuous. A total of 5% of the respondents (n = 33) indicated that they would perform a physical examination (brief screening or a complete functional analysis) themselves but would then refer the patient to a specialized colleague.

**Figure 1:**
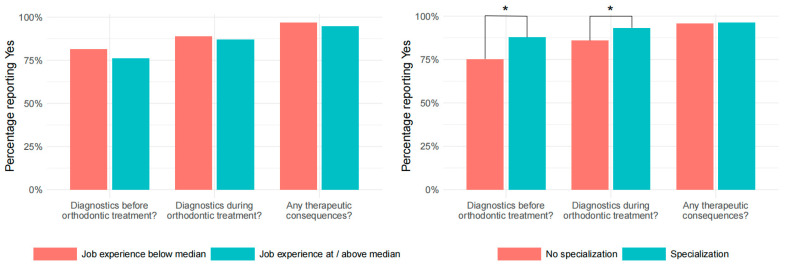
Management of TMD-related diagnostics before and during orthodontic treatment, and the resulting therapeutic consequences—differences in approach with regard to the job experience (**left**) and specialization of practitioners (**right**). * *p*-value < 0.05.

An analysis of the affirmative answers regarding all diagnostics that take place before orthodontic therapy is shown separately according to the level of professional experience and the level of specialization in Figure 1 (see Figure 1).

### 3.2. Diagnostics in the Course of Orthodontic Therapy

With regard to a further course of therapy, 33% of the respondents (n = 204) stated that they perform a complete functional analysis at least once during orthodontic therapy, irrespective of the initial findings. Most of the responding specialist dentists, namely 56.6% (n = 350), answered that they only perform a complete functional analysis during orthodontic therapy if the initial findings were positive. A total of 10.4% of the respondents (n = 64) reported that they generally do not perform a complete functional analysis during orthodontic treatment.

An analysis of the affirmative answers regarding all diagnostics that take place in the course of orthodontic therapy is shown separately according to the level of professional experience and the level of specialization in Figure 1 (see Figure 1).

### 3.3. Therapeutic Consequences

A total of 60.1% of the respondents (n = 370) stated that pre-therapeutically diagnosed, non-painful temporomandibular joint clicking (without further positive findings) has an influence on their planning of orthodontic treatment. In the case of TMD symptoms, 38.3% (n = 232) of the respondents offer pre-orthodontic therapy in their own practice, while 21.3% (n = 129) always refer patients to a cooperating specialist. A total of 35% of the respondents (n = 212) stated that they only refer patients to a specialist in complex cases. Another 4.3% (n = 26) stated that they start orthodontic therapy regardless of the detected TMD symptoms.

An analysis of the affirmative responses to the consequences drawn from TMD-related diagnostics is presented separately according to the level of professional experience and the level of specialization in Figure 1 (see Figure 1).

### 3.4. Influence of Work Experience and Specialization in the Field of TMDs

Table 1 and Table 2 show differences in clinical procedures between responding orthodontists with varying levels of professional experience and TMD-related specialization. The clinical procedures include details on diagnostics before and during orthodontic therapy, as well as therapeutic decisions derived from the findings.

There is no evidence that orthodontists with greater professional experience follow different procedures than those with less experience, with one exception. No statistically significant differences were found between the two groups in whether they conduct a brief screening (Table 1, *p* = 0.072; odds ratio = 0.725) or a complete functional analysis prior to orthodontic therapy (Table 1, *p* = 0.912; odds ratio = 1.029). The decision to perform a complete functional analysis after an initially conspicuous screening is made slightly more often by experienced orthodontists, but this difference is also statistically insignificant (Table 1, *p* = 0.452; odds ratio = 0.874). Further differences regarding clinical procedures according to reported symptoms, attention to non-painful TMJ clicking, referral in case of abnormalities, and the decision not to perform any TMJ examinations fail to show statistically significant differences between orthodontists with less or more than 25 years of professional experience (Table 1, *p* > 0.05). Only the decision to realize TMD therapy before orthodontic therapy in their own practice showed differences between the groups. In the group with greater professional experience, it was stated that TMD therapy was offered statistically significantly more often in their own practice (Table 1, *p* = 0.030; odds ratio = 1.447).

Specialization in the field of TMDs is statistically significantly associated with the likelihood of conducting a complete functional analysis prior to orthodontic therapy (Table 2, *p* < 0.001; odds ratio = 2.680). An examination depending on patient-reported symptoms is initiated statistically significantly less often among specialized orthodontists (Table 2, *p* = 0.008; odds ratio = 0.522). TMJ clicking seems to play a statistically significantly greater role in specialized orthodontists (Table 2, *p* = 0.039; odds ratio = 1.495). They refer patients to a colleague statistically significantly less often for symptoms and abnormal findings than non-specialized orthodontists (Table 2, *p* < 0.001; odds ratio = 0.217), and they more often treat TMDs themselves/in their own practice (Table 2, *p* < 0.001; odds ratio = 3.377). An additional complete functional analysis in the course of orthodontic treatment, either as a matter of principle or due to an abnormal initial finding, is also performed statistically significantly more frequently by specialized orthodontists (Table 2, *p* = 0.012; odds ratio = 2.275).

Figure 1 provides an overview of the answers given concerning different procedures before and during orthodontic treatment, as well as the therapeutic consequences that can be drawn from TMD-related findings, with stratification according to professional experience and specialization. The group of less experienced practitioners (less than 25 years since licensure) did not differ statistically significantly from the more experienced practitioners in their answers to the question of whether they perform either a brief screening or a complete functional analysis before orthodontic treatment without the patient having previously been conspicuous (diagnostics before orthodontic treatment? Figure 1, *p* > 0.05). The same applies to the question of TMD-related diagnoses during orthodontic treatment (diagnosis during orthodontic treatment? Figure 1, *p* > 0.05). There was no statistically significant difference in the responses between the two groups when asked about therapeutic consequences, such as adjustment of the treatment plan in the case of TMD clicking, referral to a specialist, or TMD therapy before starting treatment (any therapeutic consequences? Figure 1, *p* > 0.05).

Among specialists, a statistically significantly higher percentage of respondents indicated that they perform TMD-related diagnostics before orthodontic treatment (diagnostics before orthodontic treatment? Figure 1, *p* < 0.001; odds ratio = 2.356). A statistically significantly higher proportion of specialists perform an examination during orthodontic treatment than the group of non-specialists (diagnosis during orthodontic treatment? Figure 1, *p* = 0.012; odds ratio = 2.275). The attitude toward therapeutic consequences of TMD-related findings did not differ between specialists and non-specialists (any therapeutic consequences? Figure 1, *p* > 0.05).

## 4. Discussion

In a meta-analysis, the worldwide average prevalence of TMDs was determined to be 34% and 31% [1]. For Europe, the prevalence was found to be 29%. A prevalence of 11% was shown among children and adolescents [25]. These data show that TMD is a widespread disease. Considering these figures in the context of the results of the present study, standardization of diagnostic tools in the examination of functional aspects of the stomatognathic system is required, and not only for research reasons.

There are only a few studies that provide evidence-based insights into the management of TMDs before and during orthodontic therapy. The lack of specific recommendations for clinical procedures may impede orthodontists in their comprehensive treatment at the expense of the orthodontic patient. Despite this lack of formal guidance, the results of this survey show that orthodontists in Germany are concerned with TMD-related risks and are willing to invest time in TMJ examinations prior to orthodontic therapy.

As with any questionnaire-based study, bias in the results cannot be ruled out. A selection bias could be due to the fact that orthodontists who consider TMD-related diagnostics and therapy to be particularly relevant are more inclined to participate in such a study. Our sample shows a slight over-representation of experienced practitioners (with a mean of 25 years) compared to the overall population of orthodontists in Germany (mean of 19 years; data from the Federal Dental Association). Both increased interest in TMDs and above-average professional experience may have influenced the answers to the questions we asked in such a way that the diagnosis of TMDs in the context of orthodontics was assigned a greater role. Social desirability bias influences participants to be more likely to give the perceived desirable answer, especially to controversial questions. Anonymization was used to try to minimize this influence.

The response rate achieved was comparatively high in comparison with other similar studies conducted in Germany [26,27]. As no personal data were collected as part of this study and the questionnaires were sent by post, there was no possibility of reminding practices that had not responded within the specified period of time. Unfortunately, for this reason, it was not possible to extend this study with a follow-up survey. After evaluating the initial results, questions arose that would require a second wave of the survey at a later date. Therefore, the complete anonymization of the participants must be considered a shortcoming of the study design. Future studies should address this issue by using a pseudonymized online survey.

Participating orthodontists who answered the questionnaire—irrespective of their own level of professional experience or specialization—agreed on the importance of a brief screening for TMDs as a component of the initial orthodontic evaluation process. Approximately two-thirds of all responding orthodontists stated that they always perform screening for TMDs prior to orthodontic therapy.

Whether a screening is appropriate to address TMD-related problems (instead of only a medical history or a complete functional analysis) is debatable. On the one hand, performing a screening examination is less time-consuming than a thorough physical examination. Without screening or further examinations, occlusion-changing therapy in the case of an undetected TMD would be considered a treatment error in court [28]. On the other hand, routine screening may lead to unnecessary further diagnostic steps and possibly to an overtreatment of a clinically irrelevant condition [29]. In addition, once a patient’s medical history indicates TMD-related symptoms, a complete functional analysis may be imperative, hence rendering a screening unnecessary.

Orthodontic therapy should be halted as long as patients suffer from pain indicative of TMDs. It has been shown that experimentally induced pain of the masticatory muscles significantly changes the mandibular range of motion and thus complicates orthodontic therapy [30]. Whether there is a need for a complete examination of the stomatognathic system with regard to its functional aspects of the TMJ cannot currently be answered in an evidence-based manner. However, for forensic reasons, a brief screening of the TMJ protects the practitioner from legal consequences in the case of TMD symptoms occurring during or after orthodontic therapy [28]. Only 2.5% of the responding participants reported not performing a TMD-related examination prior to orthodontic treatment. Whether legal reasons are responsible for this low number cannot be deduced from our data. More professional experience or specialization in the field of the TMJ showed no association in this regard.

The recommendation for repeated complete functional analyses during orthodontic treatment is narrow and limited to patients who develop symptoms [31]. A correct diagnosis with subsequent interruption of orthodontic therapy and simultaneous treatment of TMD symptoms is crucial. A total of 87% of the respondents reported conducting a complete functional analysis at least once during the course of orthodontic therapy. In the group of responding orthodontists who were specialized in the field of TMDs, the percentage was even higher (93%). Breaking these numbers down further, we see that a relatively high percentage (33%) do this regardless of the initial findings. In the cost–benefit analysis, this approach could be discussed critically.

It is unclear whether recurrent painless clicking in the temporomandibular joint should be considered pathological, or whether its presence is associated with an increased risk of TMDs. Recent studies show that no such correlation exists and that TMJ clicking can be considered a “harmless variation of normality” [29,32]. In fact, a large-scale study showed that among patients aged 20–81 years, 20% had TMJ clicking in at least one temporomandibular joint. However, restricted maximum mouth opening with or without pain was diagnosed in only about 9%, pain in the TMJ in 2.7%, and pain in the masticatory muscles in 1.3% of the study participants [33]. Investigating occlusal features and TMJ clicking, Olliver and colleagues found a correlation between the presence of malocclusion and the presence of clicking [34]. Our results reveal that for 60.1% of the respondents, TMJ clicking has an influence on their planning of orthodontic treatment. With regard to the lack of correlation between TMJ clicking and TMD risks [27], this position is neither plausible nor evidence-based. Among orthodontists with specialization, this statement was statistically significantly more frequent (*p* = 0.039). Similar evidence–practice gaps have been identified in previous work, where the majority of participant dentists reported believing that for TMD symptoms, including TMJ clicking, occlusal adjustment or “selective grinding” was the best treatment option [35,36].

Nearly all responding orthodontists in this survey indicated that consequences would be incurred if a patient was found to have TMD symptoms, whether in the sense of pre-orthodontic TMD therapy in their own practice or referral to a specialist. Combined, 4.3% stated that they would start orthodontic therapy regardless of the findings, and only 2.5% stated that they generally do not perform any TMD-related examinations before orthodontics. However, this has to be seen critically. The question arises as to why a small proportion of respondents perform a TMD-related examination if their approach ultimately has no therapeutic consequences. Greater professional experience or specialization showed no statistically significant association here. Unfortunately, our results do not indicate whether experienced practitioners expect an improvement through orthodontic therapy and thus potentially downplay the importance of TMD therapy. Survey-based research in this direction should be further pursued.

This study was able to provide insight into the basic management of TMD patients in orthodontics. In addition, the type of examination performed in Germany is of great interest, e.g., whether the screening includes palpation of the joint and how pain is assessed. Beyond the scope of this study, further research is urgently needed to determine the exact examinations performed in practice. The heterogeneous approach to TMD diagnostics in Germany can be inferred from the information on which protocols are used. Only 1.4% (n = 9) of respondents reported using the RDC/TMD or DC/TMD, and only 7.8% (n = 49) reported using a pain or depression questionnaire (data not shown). Once again, the disadvantage of the multiple heterogeneous protocols used in Germany is evident and should be addressed through targeted information and training.

## 5. Conclusions

German orthodontists are usually very aware of their responsibility for TMD-related diagnostics. The handling of screening or complete functional analysis and TMD therapy in the context of orthodontic treatment varies among practitioners, with a discrepancy between current scientific insights and established clinical procedures. The evidence–practice gap in the field of TMDs and the inconsistency in TMD-related standard procedures are problems that exist beyond orthodontics and should be addressed in dentistry more generally. Neither longer professional experience nor the functional diagnostic specialization of German orthodontists led to a more evidence-driven approach.

Our results show heterogeneity not only in management but also, according to some initial indicators, in the details of diagnostics and therapy per se. Further studies should be carried out to investigate imaging, the type of splint therapy, applied protocols, and their success in the context of orthodontic treatment in Germany.

## Figures and Tables

**Table 1:** Differences in diagnostic and therapeutic approaches with respect to orthodontists’ professional experience.

	Professional Experience Below Median			Professional Experience At/Above Median						Difference
	n	Total	Rel. Freq.	n	Total	Rel. Freq.	OR	CI Lower	CI Upper	*p*-Value
In general, perform a brief screening	205	290	0.71	208	327	0.64	0.725	0.509	1.031	0.072
In general, perform a complete functional analysis	45	290	0.16	52	327	0.16	1.029	0.652	1.631	0.912
Complete functional analysis if findings in screening	112	290	0.39	116	327	0.35	0.874	0.621	1.229	0.452
Complete functional analysis if patient reports symptoms	57	290	0.20	74	327	0.23	1.195	0.796	1.800	0.377
No functional examination	7	290	0.02	8	327	0.02	1.014	0.317	3.329	1.000
TMJ clicking has an influence on treatment planning	172	284	0.61	192	319	0.60	0.984	0.700	1.383	0.934
Referral if reported symptoms or findings	48	290	0.17	38	327	0.12	0.663	0.407	1.075	0.082
TMD therapy before orthodontic treatment in symptomatic patients	268	289	0.93	294	324	0.91	0.768	0.407	1.425	0.384
TMD therapy before orthodontic treatment offered in own practice	94	290	0.32	134	327	0.41	1.447	1.027	2.043	0.030 *
Additional examination during orthodontic treatment (always or conditional on initial examination)	258	290	0.89	285	327	0.87	0.842	0.498	1.412	0.536

Note: * *p*-value < 0.05. OR = odds ratio; CI lower = lower bound of 95% confidence interval; CI upper = upper bound of 95% confidence interval.

**Table 2:** Differences in diagnostic and therapeutic approaches with respect to orthodontists’ specialization in the field of TMDs.

	No Specialization			Specialization						Difference
	n	Total	Rel. Freq.	n	Total	Rel. Freq.	OR	CI Lower	CI Upper	*p*-Value
In general, perform a brief screening	310	462	0.67	111	164	0.68	1.027	0.692	1.536	0.923
In general, perform a complete functional analysis	54	462	0.12	43	164	0.26	2.680	1.665	4.302	<0.001 *
Complete functional analysis if findings in screening	163	462	0.35	67	164	0.41	1.267	0.863	1.853	0.221
Complete functional analysis if patient reports symptoms	110	462	0.24	23	164	0.14	0.522	0.305	0.865	0.008 *
No functional examination	15	462	0.03	1	164	0.01	0.183	0.004	1.209	0.083
TMJ clicking has an influence on treatment planning	263	456	0.58	106	158	0.67	1.495	1.007	2.237	0.039 *
Referral if reported symptoms or findings	79	462	0.17	7	164	0.04	0.217	0.082	0.482	<0.001 *
TMD therapy before orthodontic treatment in symptomatic patients	422	459	0.92	150	163	0.92	1.012	0.509	2.133	1.000
TMD therapy before orthodontic treatment offered in own practice	136	462	0.29	96	164	0.59	3.377	2.299	4.983	<0.001 *
Additional examination during orthodontic treatment (always or conditional on initial examination)	397	462	0.86	153	164	0.93	2.275	1.152	4.913	0.012 *

Note: * *p*-value < 0.05. OR = odds ratio; CI lower = lower bound of 95% confidence interval; CI upper = upper bound of 95% confidence interval.

## Data Availability

This study was registered in the German Clinical Trials Registry. All collected data can be viewed at https://www.drks.de/search/de/trial/DRKS00022090/details (accessed on 16 September 2024).

## Appendix A

### Questionnaire

1. How long have you been practicing as an orthodontist? Year
2. In which year did you obtain your dental license? Year
3. Does your practice have a focus on temporomandibular disorders (TMDs—diagnostics and therapy)? Yes/No
4. Have you acquired one or more additional qualifications in the field of TMDs (diagnostics/therapy)? Yes/No
5. Please mark the statement that applies to you. Before orthodontic therapy…
  - I always conduct a brief TMD-related screening
  - I always conduct a complete functional analysis
  - I only conduct a complete functional analysis if the findings of the brief screening were conspicuous
  - I only perform a complete functional analysis if the patient expresses TMD symptoms during the consultation or if there is evidence of them in the written medical history
  - In the case of a positive medical history and/or positive screening results, I always refer the patient to a colleague specialized in the field of TMDs
  - I do not perform any TMD-related examinations
6. Please mark the statement that applies to you. During active orthodontic therapy…
  - I conduct a complete functional analysis independently of the initial findings (at regular intervals)
  - I only perform a complete functional analysis if the initial findings are positive
  - I never perform a complete functional analysis
7. Does pre-therapeutically diagnosed, non-painful temporomandibular joint clicking (without further positive findings) have an influence on your orthodontic treatment planning? Yes/No
8. Please choose the applicable statement. In the case of TMD symptoms/signs…
  - I always perform the pre-orthodontic TMD therapy
  - I always refer the patient to a cooperating TMD specialist
  - I only refer the patient to a cooperating TMD specialist if the case is complex
  - I start orthodontic therapy independently of the TMD-related findings
